# Development of an Instrument to Measure Resilience to Misinformation on Social Media: Measurement Properties and Validation

**DOI:** 10.2196/72449

**Published:** 2025-09-10

**Authors:** Rafaela Rosário, Silvana Martins, Tina D Purnat, Elisabeth Wilhelm, Cláudia Augusto, Maria José Silva, Juliana Martins, Ana Duarte

**Affiliations:** 1 School of Nursing University of Minho Braga Portugal; 2 Health Sciences Research Unit: Nursing Escola Superior de Enfermagem de Coimbra Coimbra Portugal; 3 Research Centre on Nursing University of Minho Braga Portugal; 4 ProChild CoLAB Against Poverty and Social Exclusion Association Guimarães Portugal; 5 Harvard TH Chan School of Public Health Harvard University Boston, MA United States; 6 University of West Attica Athens Greece

**Keywords:** resilience, misinformation, social media, instrument, psychometric analysis, exploratory factor analysis, confirmatory factor analysis

## Abstract

**Background:**

The spread of misinformation on social media poses significant risks to public health and individual decision-making. Despite growing recognition of these threats, instruments that assess resilience to misinformation on social media, particularly among families who are central to making decisions on behalf of children, remain scarce.

**Objective:**

This study aimed to develop and evaluate the psychometric properties of a novel instrument that measures resilience to misinformation in the context of social media among parents of school-age children.

**Methods:**

The development process followed a multistep approach, including item generation based on relevant frameworks, expert review for content validation, and field testing. The psychometric properties were assessed using data from 511 Portuguese parents (418/511, 82.6% women; mean age 41.2, SD 6.1 y). The dataset was randomly divided into 2 subsamples. An exploratory factor analysis was conducted with the first subsample, followed by confirmatory factor analysis with the second subsample. The internal consistency was evaluated using Cronbach α and McDonald ω.

**Results:**

On the basis of the findings from the literature review, an initial pool of 18 items was created to reflect key dimensions of resilience to misinformation. Following expert review, the items were refined to a 15-item scale, which was disseminated for pilot and preliminary testing. A 14-item instrument emerged with a 2-factor structure: (1) stress resistance and resilience to misinformation (5 items) and (2) self-control regarding misinformation (9 items). The confirmatory factor analysis supported the model’s fit, and both factors demonstrated acceptable reliability (Cronbach α=0.73; McDonald ω=0.69).

**Conclusions:**

The resilience to misinformation on social media instrument demonstrates acceptable psychometric properties and offers valuable insight into how parents engage with and respond to web-based misinformation. This tool may support public health efforts by identifying vulnerable populations and informing the development of targeted strategies to enhance resilience to misinformation within family contexts.

## Introduction

The widespread use of social media has significantly transformed the health information landscape, introducing both opportunities and substantial risks. In particular, social media has become a major conduit for the rapid spread of health misinformation—false or misleading health-related content—which poses serious challenges to public health [1,2]. The volume, speed, and often persuasive nature of online content have made it increasingly challenging for individuals to discern credible information, especially during public health crises [3]. This has led to a phenomenon termed the *infodemic*, characterized by information overload, contradictory or outdated messages, and information voids that can result in confusion, disengagement, and risky behaviors [4,5]. Such patterns not only compromise individual health decisions but also erode social cohesion, trust in health professionals, and adherence to evidence-based recommendations, ultimately prolonging disease cycles and perpetuating harmful health behaviors [6].

Among those affected by the infodemic and misinformation are individuals who search for health information on behalf of others, a group that includes caregivers, educators, and most notably, parents [7]. Parents of school-age children represent a particularly important population in this context. Children, especially those aged 6 to 10 years, are at the early stages of formal education and cognitive development, are increasingly exposed to digital environments, and often lack the necessary skills to evaluate online content [8]. In these formative years, children depend heavily on their parents to navigate, interpret, and mediate the health information they encounter. At the same time, parents themselves frequently face uncertainties and information gaps, often exacerbated by the overwhelming volume and variable quality of content shared through social media [7]. Therefore, the ability of parents to critically assess health information is not only crucial for their decision-making but also for shaping the health literacy and behaviors of their children.

The current challenges underscore the urgent need to enhance digital health literacy and to foster individual resilience to misinformation. This refers to the capacity to critically evaluate, resist, and recover from exposure to false or misleading information [9,10]. Despite growing interest in strategies to reduce belief in and the spread of misinformation, such as redirecting attention to verified content or implementing systemic countermeasures [9-12], individual-level resilience remains an underdeveloped focus in both research and intervention efforts [1,10]. Resilience has long been studied across disciplines as a multifaceted construct [13]. It has been conceptualized as a dynamic and malleable process, a stable individual trait, and an outcome that emerges following exposure to adversity [14]. It broadly describes how individuals draw upon internal and external resources to manage challenges and adapt positively to difficult circumstances, thereby fostering a sense of hope and future orientation [15,16]. Contemporary views increasingly define resilience as a dynamic process shaped by the interplay of individual characteristics (eg, cognitive flexibility and emotional regulation) and socioenvironmental influences (eg, social support and community context) [14,17]. It is not simply the absence of risk factors but the presence of protective factors that promote positive adaptation in the face of adversity [13-15,18,19]. Resilience is associated with critical capacities, such as stress regulation and self-control [19,20], and has been examined across multiple ecological levels, including individual, familial, community, and cultural contexts [13].

This study adopts a reconceptualization of resilience, considering current public health challenges, viewing it as a context-dependent process shaped by stressors [14-16]. Within the domain of misinformation, such stressors include constant exposure to confusing, unreliable, or harmful content online. Understanding resilience in this context is key to designing effective interventions that empower individuals to filter, assess, and reject misinformation.

However, to date, no validated instrument exists to specifically measure resilience to misinformation disseminated via social media, particularly among parents of school-age children. Addressing this gap is essential to identifying vulnerable populations and tailoring interventions that enhance both parental and family-level resilience to misinformation. In this study, we aim to develop and evaluate the psychometric properties of a novel instrument that measures resilience to misinformation in the context of social media, specifically targeting parents of school-age children.

## Methods

### Instrument Development

The development of the resilience to health misinformation in the context of social media instrument followed standardized criteria (eg, selecting and formulating items, scoring issues, pilot testing, and field testing) [21], allowing the development of a theoretically grounded and context-specific measure of individual resilience to misinformation encountered in digital environments, particularly social media.

The formulation of items was based on the examination of the resilience instruments [18,19,22], which helped identify relevant domains and behavioral expressions of resilience. Drawing from these sources, we identified core competencies and attitudes relevant to critically evaluating and resisting misinformation, including cognitive reflection (eg, I think carefully before I create a new post), emotional self-regulation (eg, I easily give up on seeing or reading the content that is on social media), and personal agency (eg, I like to ensure that my posts on social media are not dubious). These dimensions were operationalized into an initial pool of 18 candidate items, written in plain language and tailored to reflect everyday social media experiences. Each item was scored on a 5-point Likert scale: (1) strongly disagree, (2) disagree, (3) neutral, (4) agree, and (5) strongly agree.

To ensure content validity and appropriateness for the target population, a panel of 5 experts was convened. The panelists were selected through a structured nomination process based on their expertise in communication, psychology, and health, along with input from parents of school-age children to incorporate lived experience into the review.

The panel was asked to evaluate each item for comprehensibility, relevance, and completeness. The review process included 2 iterative rounds: in the first round, panelists provided individual ratings and comments on each item via a web-based survey; in the second round, a synchronous discussion was held to reach consensus on the proposed revisions. Because of this process, 3 items were deemed redundant or unclear and were removed, while 5 additional items were reworded to improve precision and reduce ambiguity.

Following the expert review, the revised 15-item version was pilot-tested with 15 participants from the target population—parents of school-age children (aged 6 to 10 years). The participants were recruited through local parent associations using a purposive sampling approach to ensure variation in education level and digital media use.

The participants were asked to complete the instrument and provide feedback regarding item clarity, interpretability, and response burden. Feedback was collected through brief follow-up interviews. This user-centered pilot test allowed the identification of minor structural and linguistic adjustments, which were incorporated into the final version of the instrument. No items were removed during the pilot phase, although adjustments were made to the wording of 3 items to improve clarity and alignment with the reading level of a general adult population.

The final instrument comprised 15 items. Each item contributes equally to the total score, which ranges from 1 (indicating low resilience or high susceptibility to misinformation) to 5 (indicating high resilience or low susceptibility to misinformation). Items 6, 9, and 11 to 15 were negatively worded and thus reverse-coded before computing the composite score. Higher scores indicate greater resilience to misinformation on social media.

The instrument was originally developed in European Portuguese. For this publication, the items were translated into English using a forward and backward translation process, following the best practices recommended in the literature to ensure conceptual and linguistic equivalence (Multimedia Appendix 1) [23].

### Study Design and Participants

This study was embedded within the BeE-school project (clinical trials registration: NCT05395364), a pragmatic cluster randomized controlled trial conducted in the north of Portugal and designed to evaluate the effectiveness of a school-based intervention on children’s health [24].

For the present psychometric evaluation, a cross-sectional study design was used, based on the baseline data from parents participating in the trial. Eligible participants met the following criteria: (1) were Portuguese speaking, (2) were aged ≥18 years, (3) were a parent or legal guardian of a school-age child (aged 6-10 years), and (4) provided consent to participate in the baseline data collection.

All parents of children enrolled in the trial (N=735) were invited to complete a structured, paper-based questionnaire, which included the newly developed resilience to misinformation in the context of social media instrument. A total of 511 parents completed the questionnaire, yielding a response rate of 69.7% (511/735).

### Sampling Technique

A nonprobabilistic convenience sampling approach was applied within the context of the larger trial. The schools participating in the BeE-school project facilitated the distribution of the questionnaires to families, and the completed surveys were returned via sealed envelopes through the schools. This strategy enabled access to a diverse sample of parents from the local community.

### Participants

Among the respondents, the majority (418/511, 82.6%) were mothers, with a mean age of 41.2 (SD 6.1) years. Most participants were married or cohabiting (392/511, 78.1%), had completed university-level education (219/511, 44.9%), and were employed at the time of the study (390/511, 81.4%). The sociodemographic characteristics of the sample are presented in Table 1.

**Table 1:** Sample characterization.

Characteristics		Values
Mother, n (%)		418 (82.6)
Father, n (%)		79 (15.6)
Other, n (%)		9 (1.8)
**Level of schooling, n (%)**		
	Basic education	82 (16.8)
	Secondary education	187 (36.7)
	Higher education	219 (44.9)
**Marital status, n (%)**		
	Married or cohabiting	392 (78.1)
	Divorced	43 (8.6)
	Single	62 (12.4)
	Widower	5 (1)
**Employment status, n (%)**		
	Employed	390 (81.4)
	Unemployed	88 (18.4)
	Retired	1 (0.2)
Age (y), mean (SD)		41.2 (6.1)

### Ethical Considerations

This study was approved by the ethics committee for Life and Health Sciences of the University of Minho (CEICVS 009/2022) and was conducted in accordance with the Declaration of Helsinki for human studies. The study implemented rigorous measures to protect the collected data (eg, General Data Protection Regulation) and minimize risks associated with data processing, including pseudonymization and restricted-access control to ensure confidentiality. Before data collection, parents provided written informed consent. No compensation was provided to participants.

### Statistical Analysis

**Figure 1:**
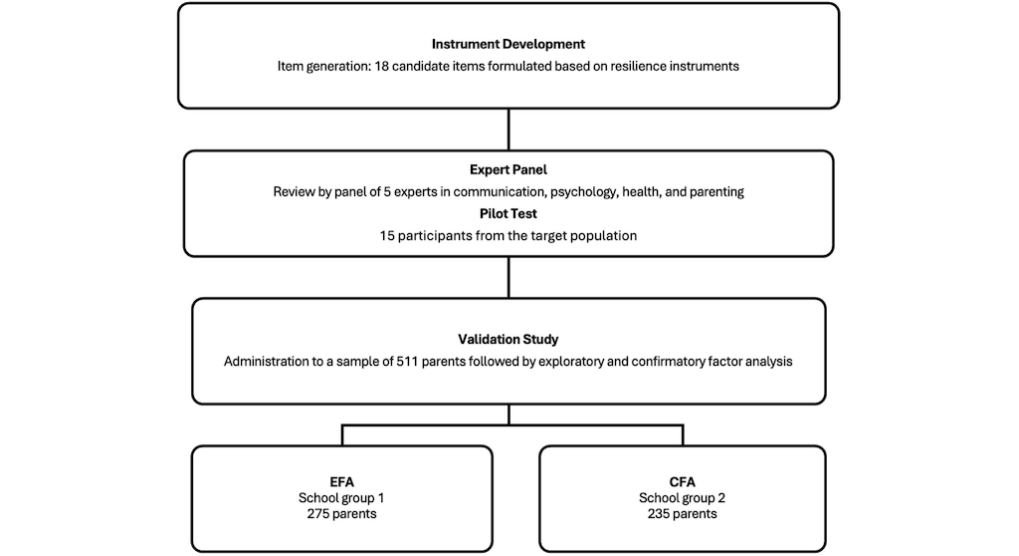
Development and validation process of the resilience to misinformation on social media instrument. CFA: confirmatory factor analysis; EFA: exploratory factor analysis.

Data analysis was performed using SPSS software (version 29.0; IBM Corp), Factor 9.2 (Lorenzo-Seva & Ferrando, Rovira i Virgili University) [25], and JASP (version 0.18.1.0; JASP Team, University of Amesterdam). A level of significance of .05 was considered. The analysis followed the COSMIN (Consensus-Based Standards for the Selection of Health Measurement Instruments) and COMET (Core Outcome Measures in Effectiveness Trials) guidelines on the taxonomy of measurement properties [26]. Initial data screening revealed no missing values. However, a few moderate univariate outliers were identified using Mahalanobis distance [27]. These cases were retained in the dataset because of their limited influence on the overall results. To ensure robust model testing, the sample was divided into 2 distinct subsamples based on 2 pre-established groupings of schools participating in the study. The parents from group 1 schools completed the version of the questionnaire used for the exploratory factor analysis (EFA), while parents from group 2 schools completed the version used for the confirmatory factor analysis (CFA; Figure 1).

Psychometric sensitivity was assessed through the descriptive statistical analysis of item response distributions, focusing on central tendency, skewness, and kurtosis. Items were flagged as potentially problematic if they showed skewness >3 and kurtosis >7 [28].

The EFA was conducted on the first subset to explore the factor structure of the resilience to misinformation on social media questionnaire. Analyses were based on a polychoric matrix, using robust diagonally weighted least squares as the extraction method [29]. The number of factors was determined via the parallel analysis technique with random permutation of the observed data [30], and the rotation method used was robust promin [31]. The model fit was assessed using the root mean square error of approximation (RMSEA), comparative fit index (CFI), and Tucker-Lewis Index (TLI), with the accepted thresholds being RMSEA<0.08 (95% CI not exceeding 0.10), and CFI and TLI ≥0.90 [32].

Factor stability was assessed using the H-index. Values >0.80 indicated a well-defined latent structure, suggesting that the factor would likely remain stable across different samples [33]. The CFA was then conducted on the second subset to confirm the factor structure derived from the EFA. Three models with different configurations were tested: (1) a unidimensional model, (2) a 2-factor model from the EFA, and (3) a refined 2-factor model with residual covariances based on modification indexes.

The model fit was assessed using the chi-square test, the χ^2^ to df ratio (χ^2^/df), the CFI, the TLI, and the RMSEA. Acceptable model fit was defined for χ^2^/df <5, CFI and TLI ≥0.90, and RMSEA <0.08 [32]. The best-fitting model was visually represented using Structural Equation Modeling diagnostic tool [34,35].

To establish the factorial validity, the standardized factorial weights were examined. Items with loadings ≥0.50 (λij ≥0.50 and λij^2^ ≥0.25) were considered valid indicators of their respective constructs [27]. The convergent validity was evaluated using the average variance extracted (AVE) [36-38], with values >0.50 indicating adequacy [36]. The discriminant validity was assessed by computing the heterotrait-monotrait ratio of correlations (HTMT), with values <0.90 suggesting acceptable discriminant validity [39].

Finally, the internal consistency was measured using Cronbach α and McDonald ω. Values ≥0.70 were considered acceptable, indicating a good level of internal consistency [40]. The interitem reliability was measured by computing the mean interitem correlations, which were considered acceptable within the range of 0.15 to 0.50 [41]. The corrected item-total correlations were also analyzed, with acceptable thresholds set at ≥0.20 [27]. The Pearson correlation coefficients among the dimensions of the instrument were interpreted as follows: >0.80 (very strong), 0.60 to 0.80 (strong), 0.40 to 0.60 (moderate), 0.20 to 0.40 (weak), and <0.20 (negligible) [42].

## Results

### Study 1: Item Generation and Content Validation

This initial phase involved developing the item pool, evaluating item relevance, and refining item wording based on expert feedback and pilot testing. The result was a 15-item instrument reflecting two conceptually grounded dimensions: (1) stress resistance and resilience to misinformation and (2) self-control regarding misinformation.

### Study 2: EFA Sample

#### Preliminary Analysis

In Table 2, we present the resilience misinformation on social media instrument items’ descriptive statistics. Skewness and kurtosis values were within acceptable ranges, supporting the psychometric sensitivity of the items. The 5-point Likert-type scale was entirely used in 73% of the items in the EFA sample, and the average scores ranged from 2.99 (SD 1.36) for item 12 to 4.59 (SD 0.62) for item 1. It is noteworthy that these scores did not deviate significantly from the range of item medians, which had a central value ranging from 3 to 5.

**Table 2:** Descriptive and item analyses of the exploratory factor analysis sample (n=275).

Items	Values, mean (SD)	Values, median (range)	Skewness	Kurtosis
1. I am careful with (...) on social media.	4.59 (0.62)	5 (2-5)	−1.45	1.79
2. I certify (...) on social media is credible.	4.42 (0.69)	5 (2-5)	−0.98	0.54
3. I easily give up (...) that are on social media.	3.53 (1.07)	4 (1-5)	−0.23	−0.90
4. I control (...) on social media.	4.55 0.62)	5 (2-5)	−1.24	1.37
5. I think carefully (...) comment on social media.	4.53 (0.67)	5 (2-5)	−1.50	2.28
6. I publish (...) the first thing that comes to my mind.	4.35 (1.01)	5 (1-5)	−1.75	2.55
7. I like (...) are not dubious.	4.46 (0.69)	5 (1-5)	−1.56	4.15
8. I stop (...) content on social media.	4.51 (0.75)	5 (1-5)	−2.12	6.20
9. I often (...) thinking about them.	4.51 (0.86)	5 (1-5)	−2.05	4.17
10. I am calm (...) sometimes cause me.	3.98 (1.01)	4 (1-5)	−0.81	0.03
11. I get nervous (...) on social media.	3.88 (1.12)	4 (1-5)	−0.80	−0.17
12. I worry (...) on social media.	2.99 (1.36)	3 (1-5)	−0.17	−1.22
13. I am afraid (...) on social media.	3.22 (1.33)	3 (1-5)	−0.03	−1.18
14. I am easily (...) on social media.	3.43 (1.22)	3 (1-5)	−0.27	−0.93
15. I often (...) I read or see on social media.	3.62 (1.15)	4 (1-5)	−0.42	−0.75

The 15 items exhibited absolute values of skewness and kurtosis within the accepted limits for a normal distribution [28]. Specifically, skewness values ranged from −2.12 to −0.03, and kurtosis values ranged from −1.18 to 6.20. All values were within acceptable psychometric thresholds [33].

#### EFA Results

The Bartlett’s test of sphericity (2237.1_105_; *P*<.001) and the Kaiser-Meyer-Olkin value (0.781) indicated adequate sampling. Parallel analysis supported a 2-factor solution. One item (item 3) was excluded because of a loading <0.30. Final factor loadings and composite reliability indexes of the items are presented in Table 3. The composite reliability of the factors exceeded 0.70 for both factors, indicating acceptable reliability. Convergent validity was supported (AVE=0.704 for factor 1; 0.562 for factor 2). Overall, the fit indexes for the instrument were deemed satisfactory (χ^2^_76_=181.7, *P*<.001; RMSEA=0.105; CFI=0.945; TLI=0.924).

**Table 3:** Factor structure of the resilience to misinformation instrument^a^.

Items	Factor 1	Factor 2
1. I am careful with (...) on social media.	—^b^	0.746
2. I certify (...) on social media is credible.	—	0.726
3. I easily give up (...) that are on social media.	—	—
4. I control (...) on social media.	—	0.804
5. I think carefully (...) comment on social media	—	0.838
6. I publish (...) the first thing that comes to my mind.	—	0.560
7. I like (...) are not dubious.	—	0.823
8. I stop (...) content on social media.	—	0.876
9. I often (...) thinking about them.	—	0.582
10. I am calm (...) sometimes cause me.	—	0.316
11. I get nervous (...) on social media.	0.733	—
12. I worry (...) on social media.	−0.845	—
13. I am afraid (...) on social media.	0.855	—
14. I am easily (...) on social media.	0.821	—
15. I often (...) I read or see on social media.	0.929	—
Composite reliability	0.90	0.922
H^c^ latent	0.937	0.924
H observed	0.963	0.932

^a^Items were retained if the loading was ≥0.30. Composite reliability and H-indexes above 0.70 indicate acceptable reliability and construct replicability.

^b^Not applicable.

^c^H-index.

### Study 3: CFA, Construct Validity, Internal Consistency, and Reliability

#### Preliminary Analysis

Table 4 presents the descriptive statistics of the resilience to misinformation on social media instrument items in the CFA sample (n=235). Skewness and kurtosis values were within acceptable ranges, supporting the psychometric sensitivity of the items. The 5-point Likert-type scale was fully used in 87% (13/15) of the items.

**Table 4:** Descriptive and item analyses of the confirmatory factor analysis sample (n=235).

Items	Values, mean (SD)	Values, median (range)	Skewness	Kurtosis
1. I am careful with (...) on social media.	4.54 (0.65)	5 (1-5)	−1.50	3.19
2. I certify (...) on social media is credible.	4.37 (0.75)	5 (2-5)	−1.09	0.83
3. I easily give up (...) that are on social media.	3.58 (1.02)	4 (1-5)	−0.41	−0.31
4. I control (...) on social media.	4.50 (0.64)	5 (1-5)	−1.41	3.63
5. I think carefully (...) comment on social media.	4.53 (0.59)	5 (2-5)	−0.96	0.62
6. I publish (...) the first thing that comes to my mind.	4.21 (1.16)	5 (1-5)	−1.46	1.09
7. I like (...) are not dubious.	4.38 (0.71)	4 (1-5)	−1.20	2.25
8. I stop (...) content on social media.	4.47 (0.73)	5 (1-5)	−2	6.11
9. I often (...) thinking about them.	4.45 (0.96)	5 (1-5)	−1.97	3.45
10. I am calm (...) sometimes cause me.	3.83 (1.04)	4 (1-5)	−0.83	0.34
11. I get nervous (...) on social media.	3.89 (1.04)	4 (1-5)	−0.64	−0.40
12. I worry (...) on social media.	2.98 (1.25)	3 (1-5)	−0.26	−1.01
13. I am afraid (...) on social media.	3.14 (1.23)	3 (1-5)	0.18	−1.08
14. I am easily (...) on social media.	3.30 (1.17)	3 (1-5)	−0.11	−0.89
15. I often (...) I read or see on social media.	3.49 (1.15)	4 (1-5)	−0.26	−0.83

The average item scores in the CFA sample ranged from 2.98 (SD 1.25) for item 12 to 4.54 (SD 0.65) for item 1. Notably, these scores did not deviate significantly from the item medians, which remained within the central value range of 3 to 5.

All 15 items exhibited absolute values of skewness and kurtosis within the acceptable limits for a normal distribution [33]. Specifically, skewness values ranged between −2.00 and −0.18, while kurtosis values ranged from −1.08 to 6.11.

**Figure 2:**
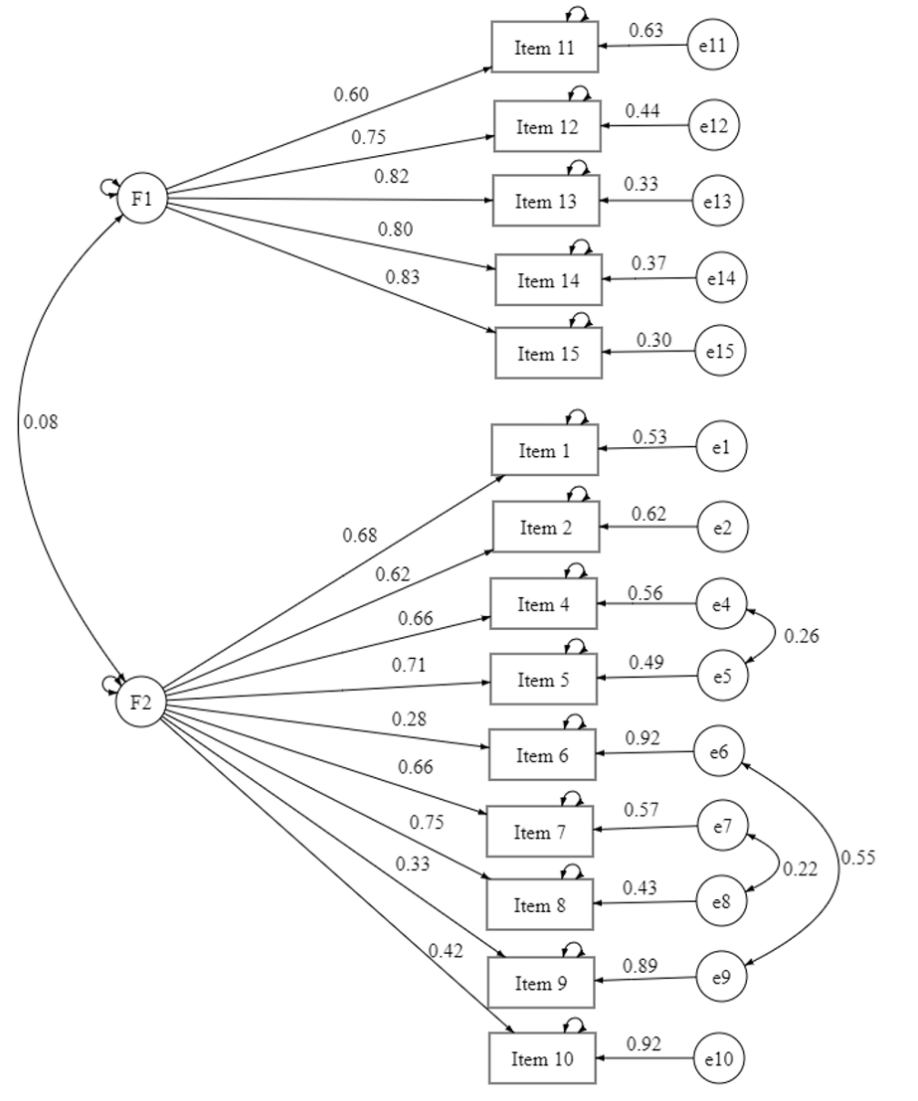
Factor loadings and covariances for 2 first-order latent factor structure. F1: stress resistance and resilience to misinformation; F2: self-control regarding misinformation.

Three models were tested (Table 5). Model 3 (modified 2-factor model) showed the best fit: χ^2^_73_=130.1, *P*<.001, CFI=0.966, TLI=0.945, and RMSEA=0.058, 95% CI 0.041-0.074 (Figure 2).

**Table 5:** Confirmatory factor analysis models fit indexes (n=235)^a^.

	Chi-square (*df*)	Chi-square/*df*	CFI^b^	TLI^c^	RMSEA^d^
Model 1	791.1 (77)	10.3	0.443	0.342	0.199
Model 2	235.1 (76)	3.09	0.876	0.852	0.094
Model 3	130.1 (73)	1.78	0.966	0.945	0.058

^a^Comparative fit index and Tucker-Lewis Index ≥0.90 and root mean square error of approximation <0.08 indicate good model fit [38].

^b^CFI: comparative fit index.

^c^TLI: Tucker-Lewis Index.

^d^RMSEA: root mean square error of approximation.

#### Construct Validity

The AVE confirmed convergent validity for factor 1 (0.585). However, it was 0.350 for factor 2. Discriminant validity was established for both factors via the HTMT (<0.90 threshold). The composite reliability was 0.874 for factor 1 and 0.817 for factor 2 [33]. The correlation between the 2 factors was weak (*r*=0.173), indicating conceptual distinction. The correlation was positive and statistically significant.

#### Common Method Variance

The Harman single-factor test, applied to all items of the instrument, revealed multiple factors, with the largest explaining 37.3% of the total variance extracted in the EFA. This outcome provides no indication of common method bias [43,44]. In contrast, the CFA model 1, in which all items loaded onto a single factor, notably failed the overall fit test (χ^2^/df=10.3, CFI=0.443, TLI=0.342, RMSEA=0.199). Consequently, we concluded that common method bias was not a significant issue in our model.

The self-control regarding misinformation factor showed a higher mean of 39.3 (SD 4.5; range 24-25). Conversely, the stress resistance or resilience to misinformation factor displayed a lower mean value of 16.9 (SD 3.1; range 8-24).

#### Internal Consistency and Reliability

Table 6 summarizes the internal consistency metrics. McDonald ω and corrected item-total correlations were acceptable across both samples. Cronbach α was <0.70 for factor 1, but the other reliability indicators (McDonald ω and the interitem correlation) supported acceptable consistency. Cronbach α was 0.672, suggesting good internal consistency evidence. In addition, we present the other reliability estimates, such as the interitem reliability and McDonald ω, allowing future comparisons with other studies (Table 6).

**Table 6:** Internal consistency of resilience to misinformation on social media instrument factors^a^.

Factors	Cronbach α	McDonald ω	MIIC^b^	CITCR^c^
Factor 1	0.333	0.715	0.113	0.444-0.714
Factor 2	0.788	0.829	0.340	0.354-0.644

^a^Acceptable thresholds: Cronbach α ≥0.70, McDonald ω ≥0.70, mean interitem correlation 0.15-0.50, corrected item-total correlation range ≥0.20.

^b^MIIC: mean interitem correlation.

^c^CITCR: corrected item-total correlation range.

In addition, the mean interitem correlations fell within the acceptable range of 0.15 to 0.50 only for factor 2 in both samples [41]. Furthermore, the corrected item-total correlations exhibited good values, surpassing 0.20 in both samples for both factor 1 and factor 2 [27].

## Discussion

### Principal Findings

This study provides satisfactory evidence regarding the overall reliability and dimensional reliability of the resilience to misinformation on social media instrument. The findings support the internal consistency of the instrument both as a whole and within its 2 distinct dimensions. Furthermore, item analysis demonstrated consistently good psychometric properties across all 14 items, with satisfactory item-total correlations and acceptable ranges of skewness and kurtosis, reflecting the precision and conceptual clarity achieved during the item development process. The interconnectedness among the items revealed a coherent structure, culminating in the identification of 2 underlying dimensions: *stress resistance and resilience* and *self-control* regarding misinformation. This dimensional structure not only enhances the comprehensiveness of the instrument, but it also aligns well with established theoretical frameworks of resilience [13-15,18].

The study supports a conceptualization of resilience to misinformation on social media as a multidimensional construct, encompassing both stable traits and dynamic states. The self-control dimension captures more trait-like characteristics [19], such as a consistent tendency to question sources, critically reflect before sharing content, and act responsibly in digital environments. These traits are generally stable over time. In contrast, the stress resistance and resilience to misinformation dimension reflects resilience as a process that focuses on emotional and psychological responses to stressors [14] triggered by misinformation exposure, including confusion, distress, or cognitive overload. These responses are context-dependent and may fluctuate based on situational factors.

Together, these dimensions provide a nuanced understanding of how parents of school-age children experience and manage misinformation. This perspective enhances the public health relevance of the instrument by enabling the design of tailored interventions that address both enduring vulnerabilities and context-sensitive reactions to misleading content.

Substantial evidence supported the construct validity and reliability of the instrument. Convergent and discriminant validity indicators were confirmed using AVE and HTMT, establishing that the 2 factors were conceptually distinct and adequately represented the underlying construct. Internal consistency was assessed using both Cronbach α and McDonald ω, acknowledging their distinct assumptions. While slight deviations were noted in α values, ω coefficients, interitem correlations, and corrected item-total correlations supported acceptable reliability.

This dual reporting allows comparability with future studies and provides a more complete picture of the performance of the scale.

The resilience to misinformation on social media instrument can serve as a valuable tool for identifying knowledge gaps in resilience to misinformation, evaluating individual susceptibility to misinformation, adversity, and uncertainty [45,46]. In addition, it enables the monitoring of the effectiveness of interventions in strengthening resilience to misinformation. By systematically gathering this information, evidence-based strategies can be developed to enhance individuals’ ability to navigate complex health information environments, resist misinformation, and adapt to uncertainty, ultimately fostering a more resilient and well-informed society.

Moreover, while the instrument was designed to assess resilience to misinformation broadly, it is important to address health misinformation, a growing public health concern [10]. A clearer understanding of resilience in this domain is crucial for designing interventions that foster critical thinking, emotional self-regulation, and responsible information-sharing behaviors, especially among parents tasked with evaluating health-related information for their children and families.

This study is not without strengths. We devised the instrument by carefully crafting items in both positive and negative formulations, thereby mitigating the susceptibility of the scale to the influence of acquiescence response bias [47,48]. This approach aligns with the recommendation of Wagnild [48] to rephrase statements and incorporate negative keying in certain items. Similarly, adjustments to the Likert scale items to improve alignment with the questions were explored to enhance their use. Still, the scale would gain value from further investigation into its test-retest reliability, as well as its predictive validity, such as through its use in longitudinal studies. It has been established that community and cultural factors play a significant role in shaping the definition and typical expression of resilience among diverse populations [49]. Therefore, it would be beneficial for future research to investigate the concept of resilience to misinformation in various cultural and population contexts, aiming to determine the stability of the construct. We used a novel approach to determining discriminant validity, demonstrating superior performance in comparison to other criteria [36] and the assessment of (partial) cross-loadings [50].

### Limitations

Despite its strengths, this study presents several limitations that warrant consideration. First, there is currently no established gold standard for assessing resilience to misinformation, which limits direct comparisons with the instrument. Future research should consider exploring convergent and discriminant validity through comparisons with related constructs, such as resilience, digital health literacy, cognitive reflection, emotional self-regulation, or critical thinking. Second, resilience to misinformation on social media instrument relies on self-reporting. While this method facilitates large-scale data collection and enhances feasibility, it introduces potential response biases, particularly those related to social desirability and acquiescence tendencies [47,51]. Such biases may affect the accuracy of reported attitudes and behaviors, especially in sensitive or socially charged domains such as misinformation. Third, the psychometric testing was conducted using baseline data from the BeE-school project, in which the vast majority of respondents were mothers (82.6%). While this group is highly relevant, given its central role in mediating and guiding children’s media use and health decisions, the predominance of female participants may limit the generalizability of findings to other caregiver profiles, particularly fathers or nonparental caregivers. Previous research indicates that women may experience higher levels of perceived stress and lower levels of self-efficacy and resilience when compared with men [52-54], which could have influenced response patterns in this study. These gender-related differences may also affect individuals’ emotional regulation, capacity to critically engage with and process information, and ability to respond to adversity [54], including exposure to misinformation on social media platforms.

Future studies should focus on construct validity, ensuring the effective functioning of the test as a construct and accurately measuring its intended aspects through well-defined items. This form of validity essentially encompasses various types of validity [55]. The examination of construct validity involves analyzing correlations between the construct under investigation, in this case, resilience to misinformation, and variables known to be interconnected [56]. Although the translation process adhered to scientifically established procedures to ensure the accuracy and equivalence of the English version, it is important to note that the instrument was developed and validated in European Portuguese. Therefore, any application in different linguistic or cultural contexts should be preceded by a thorough content analysis and followed by an independent validation process to confirm the reliability and validity of the instrument in the new setting.

While the resilience to misinformation on social media instrument demonstrated satisfactory reliability when applied to Portuguese parents of school-age children, the findings should be interpreted with caution. Further research is needed to examine the performance of the instrument across different populations, age groups, and cultural contexts. Longitudinal studies are also recommended to explore the predictive validity and stability of the scale over time. The instrument has the potential to be applied in intervention research, particularly in educational and public health contexts, to help design and evaluate strategies that foster resilience. We posit that this study contributes to the initiation of a more profound exploration of resilience to misinformation associated with social media.

Capturing resilience to misinformation in the context of social media is critical for informing public health efforts to combat the infodemic. By identifying individuals and groups, such as parents of school-age children, who may be particularly vulnerable to misleading content, this instrument provides a valuable foundation for the development of tailored interventions.

The resilience to misinformation on social media instrument demonstrated satisfactory psychometric properties and offers a promising foundation for advancing both research and practice in misinformation resilience. Continued validation and application across diverse settings will strengthen its utility and impact.

### Conclusions

The instrument demonstrated satisfactory psychometric properties, with strong evidence of construct validity, dimensional structure, and internal consistency. The 2 identified dimensions—stress resistance and resilience and self-control regarding misinformation—reflect the dynamic interplay between emotional regulation and cognitive control, and align with contemporary models of resilience. This work supports broader efforts to mitigate the impact of misinformation and promote resilience in an increasingly complex information ecosystem.

## Appendix

Multimedia Appendix 1 Resilience to misinformation on social media instrument.

## Abbreviations

AVE: average variance extracted
CFA: confirmatory factor analysis
CFI: comparative fit index
COMET: Core Outcome Measures in Effectiveness Trials
COSMIN: Consensus-Based Standards for the Selection of Health Measurement Instruments
EFA: exploratory factor analysis
HTMT: heterotrait-monotrait ratio of correlations
RMSEA: root mean square error of approximation
TLI: Tucker-Lewis Index

## Data Availability

The datasets used and analyzed during this study are available from the corresponding author on reasonable request.

## References

[1] Roozenbeek J, van der Linden S, Goldberg B, Rathje S, Lewandowsky S (2022). Psychological inoculation improves resilience against misinformation on social media. Sci Adv.

[2] Lewandowsky S, Ecker U, Cook J (2017). Beyond misinformation: understanding and coping with the “post-truth” era. J Appl Res Mem Cogn.

[3] Roozenbeek J, Schneider CR, Dryhurst S, Kerr J, Freeman AL, Recchia G, van der Bles AM, van der Linden S (2020). Susceptibility to misinformation about COVID-19 around the world. R Soc Open Sci.

[4] Zarocostas J (2020). How to fight an infodemic. Lancet.

[5] Eysenbach G (2020). How to fight an infodemic: the four pillars of infodemic management. J Med Internet Res.

[6] van der Linden S (2022). Misinformation: susceptibility, spread, and interventions to immunize the public. Nat Med.

[7] Frey E, Bonfiglioli C, Brunner M, Frawley J (2022). Parents' use of social media as a health information source for their children: a scoping review. Acad Pediatr.

[8] Shtulman A (2024). Children's susceptibility to online misinformation. Curr Opin Psychol.

[9] Roozenbeek J, van der Linden S (2019). Fake news game confers psychological resistance against online misinformation. Palgrave Commun.

[10] Roozenbeek J, van der Linden S (2022). How to combat health misinformation: a psychological approach. Am J Health Promot.

[11] Pennycook G, Epstein Z, Mosleh M, Arechar AA, Eckles D, Rand DG (2021). Shifting attention to accuracy can reduce misinformation online. Nature.

[12] Casalino N, Żuchowski I, Labrinos N, Munoz Nieto ÁL, Martín JA Digital strategies and organizational performances of SMEs in the age of coronavirus: balancing digital transformation with an effective business resilience. SSRN Journal. Preprint posted online December 1, 2019.

[13] Southwick SM, Bonanno GA, Masten AS, Panter-Brick C, Yehuda R (2014). Resilience definitions, theory, and challenges: interdisciplinary perspectives. Eur J Psychotraumatol.

[14] Smeeth D, Beck S, Karam EG, Pluess M (2021). The role of epigenetics in psychological resilience. Lancet Psychiatry.

[15] Ong AD, Bergeman CS, Bisconti TL, Wallace KA (2006). Psychological resilience, positive emotions, and successful adaptation to stress in later life. J Pers Soc Psychol.

[16] McLaughlin KA, Sheridan MA, Lambert HK (2014). Childhood adversity and neural development: deprivation and threat as distinct dimensions of early experience. Neurosci Biobehav Rev.

[17] Malhi GS, Das P, Bell E, Mattingly G, Mannie Z (2019). Modelling resilience in adolescence and adversity: a novel framework to inform research and practice. Transl Psychiatry.

[18] Windle G, Bennett KM, Noyes J (2011). A methodological review of resilience measurement scales. Health Qual Life Outcomes.

[19] Ayduk O, Mendoza-Denton R, Mischel W, Downey G, Peake PK, Rodriguez M (2000). Regulating the interpersonal self: strategic self-regulation for coping with rejection sensitivity. J Pers Soc Psychol.

[20] Hermans E, Hendler T, Kalisch R (2025). Building resilience: the stress response as a driving force for neuroplasticity and adaptation. Biol Psychiatry.

[21] de Vet H, Terwee C, Mokkink L, Knol D (2011). Measurement in Medicine: A Practical Guide.

[22] Survey on social and emotional skills: technical report. Organisation for Economic Co-operation and Development.

[23] Cantor SB, Byrd TL, Groff JY, Reyes Y, Tortolero-Luna G, Mullen PD (2005). The language translation process in survey research: a cost analysis. Hisp J Behav Sci.

[24] Martins J, Augusto C, Silva MJ, Duarte A, Martins SP, Antunes H, Novais P, Pereira B, Veiga P, Rosário R (2025). Effectiveness of a health promotion program on overweight in vulnerable children from primary schools (BeE-school): a cluster-randomized controlled trial. Int J Obes (Lond).

[25] Lorenzo-Seva U, Ferrando PJ (2006). FACTOR: a computer program to fit the exploratory factor analysis model. Behav Res Methods.

[26] Mokkink LB, Terwee CB, Patrick DL, Alonso J, Stratford PW, Knol DL, Bouter LM, de Vet HC (2010). The COSMIN study reached international consensus on taxonomy, terminology, and definitions of measurement properties for health-related patient-reported outcomes. J Clin Epidemiol.

[27] Tabachnick B, Fidell L (2019). Using Multivariate Statistics. 7th edition.

[28] Kline R (2023). Principles and Practice of Structural Equation Modeling. 5th edition.

[29] Asparouhov T, Muthen B Simple second order chi-square correction. Mplus.

[30] Timmerman ME, Lorenzo-Seva U (2011). Dimensionality assessment of ordered polytomous items with parallel analysis. Psychol Methods.

[31] Lorenzo-Seva U, Ferrando PJ (2019). Robust promin: a method for diagonally weighted factor rotation. Liberabit.

[32] Brown T (2015). Confirmatory Factor Analysis for Applied Research. 2nd edition.

[33] Ferrando PJ, Lorenzo-Seva U (2018). Assessing the quality and appropriateness of factor solutions and factor score estimates in exploratory item factor analysis. Educ Psychol Meas.

[34] Mai Y, Xu Z, Zhang Z, Yuan K (2022). An open-source WYSIWYG web application for drawing path diagrams of structural equation models. Struct Equ Modeling.

[35] Draw SEM diagrams. semdiag.

[36] Fornell C, Larcker DF (1981). Evaluating structural equation models with unobservable variables and measurement error. J Mark Res.

[37] Hair J, Black W, Babin B, Anderson R (2010). Multivariate Data Analysis. 7th edition.

[38] Valentini F, Damásio BF (2016). Variância Média Extraída e Confiabilidade Composta: Indicadores de Precisão. Psic Teor Pesq.

[39] Gold AH, Malhotra A, Segars AH (2015). Knowledge management: an organizational capabilities perspective. J Manag Inf Syst.

[40] Ventura Leon J, Caycho-Rodríguez T (2017). El coeficiente Omega: un metodo alternativo para la estimacion de la confiabilidad. Niñez y Juventud.

[41] Briggs SR, Cheek JM (2006). The role of factor analysis in the development and evaluation of personality scales. J Pers.

[42] Schober P, Boer C, Schwarte LA (2018). Correlation coefficients: appropriate use and interpretation. Anesth Analg.

[43] Podsakoff PM, MacKenzie SB, Lee J, Podsakoff NP (2003). Common method biases in behavioral research: a critical review of the literature and recommended remedies. J Appl Psychol.

[44] Podsakoff PM, MacKenzie SB, Podsakoff NP (2012). Sources of method bias in social science research and recommendations on how to control it. Annu Rev Psychol.

[45] Cajada L, Stephenson Z, Bishopp D (2023). Exploring the psychometric properties of the resilience scale. Adv Res Sci.

[46] Ahern NR, Kiehl EM, Lou Sole M, Byers J (2006). A review of instruments measuring resilience. Issues Compr Pediatr Nurs.

[47] Paulhus D, Zeigler-Hill V, Shackelford TK (2017). Socially desirable responding on self-reports. Encyclopedia of Personality and Individual Differences.

[48] Wagnild G (2009). A review of the Resilience Scale. J Nurs Meas.

[49] Ungar M (2006). Resilience across cultures. Br J Soc Work.

[50] Franke G, Sarstedt M (2019). Heuristics versus statistics in discriminant validity testing: a comparison of four procedures. Int Res.

[51] Paulhus DL (1984). Two-component models of socially desirable responding. J Pers Soc Psychol.

[52] Boardman JD, Blalock CL, Button TM (2008). Sex differences in the heritability of resilience. Twin Res Hum Genet.

[53] Özbay A, Bülbül AE (2025). The impact of psychological resilience and gender on the relationship between trauma-coping perception and levels of secondary traumatic stress in mental health workers. J Community Psychol.

[54] Vidic Z (2024). Gender differences on coping, stress, resilience and mindfulness within an academic course intervention with a mindfulness meditation component. Curr Psychol.

[55] Kline P (2000). Handbook of Psychological Testing.

[56] Campbell DT, Fiske DW (1959). Convergent and discriminant validation by the multitrait-multimethod matrix. Psychol Bull.

