# Supplementary material for: Development of an Instrument to Measure Resilience to Misinformation on Social Media: Measurement Properties and Validation
Source: J Med Internet Res. 2025 Sep 10;27:e72449. doi: 10.2196/72449 (PMC12461171; doi:10.2196/72449)
Supplement: Multimedia Appendix 1 [file jmir_v27i1e72449_app1.docx]

**Resilience to misinformation on social media instrument**

**Portuguese version**

|  | **1**  **Discordo totalmente** | **2**  **Discordo** | **3**  **Não concordo nem dicorcordo** | **4**  **Concordo** | **5 Concordo totalmente** |
| --- | --- | --- | --- | --- | --- |
| 1. Tenho cuidado com o que digo às pessoas nas redes sociais (ex. Facebook, Tik Tok, Instagram, Youtube). |  |  |  |  |  |
| 2. Certifico-me que a fonte da notícia que consulto nas redes sociais é credível. |  |  |  |  |  |
| 3. Controlo aquilo que faço nas redes sociais (ex., criar novo *post*/fazer ‘gosto’/reencaminhar ou fazer comentários nas redes sociais). |  |  |  |  |  |
| 4. Penso com cuidado antes de criar novo *post* /fazer ‘gosto’/reencaminhar ou fazer comentários nas redes sociais. |  |  |  |  |  |
| *5. Publico (ex. crio um *post* ou faço um comentário) a primeira coisa que me vem à cabeça nas redes sociais. |  |  |  |  |  |
| 6. Gosto de me certificar que as publicações que faço nas redes sociais não são duvidosas. |  |  |  |  |  |
| 7. Paro para pensar antes de publicar algum conteúdo nas redes sociais. |  |  |  |  |  |
| *8. Publico frequentemente sem pensar nas redes sociais. |  |  |  |  |  |
| 9. Sou calmo/a e aguento bem o stress que por vezes as redes sociais me provocam. |  |  |  |  |  |
| *10. Fico nervoso/a facilmente com o que leio/vejo nas redes sociais. |  |  |  |  |  |
| *11. Preocupo-me com muitas coisas que leio/vejo nas redes sociais. |  |  |  |  |  |
| *12. Tenho medo de muitas coisas que leio/vejo nas redes sociais. |  |  |  |  |  |
| *13. Fico facilmente horrorizado com o que leio/vejo nas redes sociais. |  |  |  |  |  |
| *14. Sinto-me muitas vezes nervoso/a com o que leio/vejo nas redes sociais. |  |  |  |  |  |

**English version**

|  | **1**  **Totally disagree** | **2**  **Disagree** | **3**  **Do not agree/Agree** | **4 Agree** | **5**  **Totally agree** |
| --- | --- | --- | --- | --- | --- |
| 1. I am careful with what I tell others on social media (e.g., Facebook, Instagram, Tik Tok, youtube) |  |  |  |  |  |
| 2. I certify the source of information in social media is credible. |  |  |  |  |  |
| 3. I control what I do on social media (e.g., create a new post/ do a “like”/ spread a comment on social media). |  |  |  |  |  |
| 4. I think carefully before I create a new post / do a “like”/ spread a comment on social media. |  |  |  |  |  |
| *5. I publish on social media (e.g., create a post or comment), the first thing that comes to my mind. |  |  |  |  |  |
| 6. I like to certify that my posts on social media are not dubious |  |  |  |  |  |
| 7. I stop to think before posting any content on social media. |  |  |  |  |  |
| *8. I often post content on social media without thinking about them. |  |  |  |  |  |
| 9. I am calm, and I can handle the stress that social media sometimes cause me. |  |  |  |  |  |
| *10. I get nervous easily with what I read/see on social media. |  |  |  |  |  |
| *11 I worry about many things I read/see on social media. |  |  |  |  |  |
| *12. I am afraid of many things I read/see on social media. |  |  |  |  |  |
| *13. I am easily horrified by what I read/see on social media. |  |  |  |  |  |
| *14. I often feel nervous about what I read/see on social media |  |  |  |  |  |

* Items that required reverse coding

The scores are calculated using means as follows:

Total score: all items

Stress resistance: items 10, 11, 12, 13, and 14

Self-control regarding misinformation: items 1, 2, 3, 4, 5, 6, 7, 8, and 9
